# Correlation analysis of dermal collagen and elastic fibre content between two directions of skin samples taken from chest area and its surgical implications

**DOI:** 10.1186/1749-8090-10-S1-A149

**Published:** 2015-12-16

**Authors:** Naveen Kumar, Pramod Kumar, Satheesha B Nayak

**Affiliations:** 1 Department of Anatomy, Melaka Manipal Medical College, Manipal campus, Manipal University, India, 576104; 2 Department of Plastic Surgery, King Abdul Aziz Hospital, Al Jouf, Sakaka, Saudi Arabia - 42421

## Background/Introduction

Unequal distribution of dermal collagen and elastic fibers in different orientations is reported to be one of the multi focal causes of scar related complications.

## Aims/Objectives

To study the correlation pattern between the variables of dermal collagen in horizontal (CH) and in vertical (CV) directions as well as that of dermal elastic fibers in horizontal (EH) and vertical (EV) directions.

## Method

Current research comprised of histo-morphometrical study of 120 skin samples collected in horizontal and vertical orientations from anterior chest area and lateral chest areas of formalin embalmed human cadavers (n = 30). The quantitative fraction of dermal collagen and elastic fiber content was obtained by tissue-quant image analysis. Spearman correlation coefficient (r) was computed to determine the linear association between the variables (CH/CV/EH/EV). The results were interpreted according to the degree of association after taking into consideration the significant correlation (p < 0.01 or p < 0.05) with respect to coefficient (r) values.

## Results

Significant positive correlation between CH and CV and between EH and EV were observed in both anterior (r = 0.56; r = .62) and lateral chest areas (r = .55; r = .43). However, the negative correlations were observed between CH and EH for anterior chest area (r = -.55) and between CV and EV at lateral chest area (r = -.43).

## Discussion/Conclusion

Positive correlation among dermal collagen and elastic fibers between horizontal and vertical directions, and their negative correlation within same direction could be one of the factors for the speckled behavior of scar related complications in the chest area.

